# Quality of life of women with a screen-detected versus clinically detected breast cancer in the Netherlands: a prospective cohort study

**DOI:** 10.1007/s11136-024-03783-0

**Published:** 2024-09-17

**Authors:** Abyan Irzaldy, Johannes D. M. Otten, Lindy M. Kregting, Dieuwke R. Mink van der Molen, Helena M. Verkooijen, Nicolien T. van Ravesteyn, Eveline A. M. Heijnsdijk, Annemiek Doeksen, Carmen C. van der Pol, Daniel J. Evers, Miranda F. Ernst, Ida J. Korfage, Harry J. de Koning, Mireille J. M. Broeders

**Affiliations:** 1 Department of Public Health, Erasmus MC, University Medical Centre Rotterdam, Dr. Molewaterplein 40 NA-24, Rotterdam, 3015 GD The Netherlands; 2 IQ Health Science Department, Radboud University Medical Centre, Nijmegen, The Netherlands; 3 Division of Imaging & Oncology, University Medical Centre Utrecht, Utrecht, The Netherlands; 4 Department of Surgery, St. Antonius Hospital, Utrecht, The Netherlands; 5 Department of Surgery, Alrijne Hospital, Leiderdorp, The Netherlands; 6 Department of Surgery, ZGT Hospital Group Twente, Almelo, The Netherlands; 7 Department of Surgery, Alexander Monro Hospital, Bilthoven, The Netherlands; 8 Dutch Expert Centre for Screening, Nijmegen, The Netherlands

**Keywords:** Quality of life, Breast cancer, Mass screening, Early detection of cancer

## Abstract

**Purpose:**

Breast cancer (BC) screening enables early detection of BC, which may lead to improved quality of life (QoL). We aim to compare QoL between women with a screen-detected and clinically detected BC in the Netherlands.

**Methods:**

We used data from the ‘Utrecht cohort for Multiple BREast cancer intervention studies and Long-term evaluation’ (UMBRELLA) between October 2013 and March 2022. Patients were categorized as screen-detected or clinically detected. We analysed three questionnaires, namely EORTC QLQ C-30, BR23, and HADS (Hospital Anxiety and Depression Scale) completed by BC patients shortly after diagnosis (T1) and one-year after treatment (T2). Independent t-tests were performed to compare QoL average differences between the two groups. Bonferroni-corrected *p*-value significance threshold of 0.00057 was used. The magnitude of differences was calculated using Cohen’s *d*. The clinical relevance of QLQ-C30 differences was assessed based on interpretation guideline of EORTC-QLQ-C30 results.

**Results:**

After applying inclusion and exclusion criteria, there were 691 women with screen-detected BC and 480 with clinically detected BC. Generally, screen-detected BC patients reported a better QoL. At T1, their average QLQ-C30 summary score was higher (86.1) than clinically detected BC patients (83.0) (*p* < 0.0001). Cohen’s *d* for all items ranged between 0.00 and 0.39. A few QLQ-C30 score differences were clinically relevant, indicating better outcomes in emotional functioning, general health, constipation, and fatigue for women with screen-detected BC.

**Conclusions:**

In the Netherlands, women with screen-detected BC reported statistically significant and better QoL than women with clinically detected BC. However, clinical relevance of the differences is limited.

## Abstract (plain language summary)

This study compares the quality of life (QoL) of women with breast cancer (BC) detected through screening versus through clinical detection in the Netherlands. BC screening aims to detect cancer early, potentially improving QoL. We analysed data from the UMBRELLA cohort, focusing on QoL questionnaires filled out shortly after diagnosis and one year after treatment. Results showed that women with screen-detected BC generally reported better QoL than those with clinically detected BC. For example, they had higher QLQ-C30 summary scores and several other QLQ-C30 scales and items, indicating better emotional functioning, better general health, less constipation, and less fatigue symptom. We also found that the differences in QoL are larger at one year after treatment. However, while statistically significant, the differences in QoL between the two groups were either trivial or small and may have limited clinical relevance. This study sheds light on the potential impact of mode of detection on the QoL of women with BC.

## Introduction

Breast cancer (BC) screening has been widely implemented since the 1990s. Since its implementation, a reduction in BC mortality has been observed [1–3]. Furthermore, besides reducing BC mortality, screening may lead to other health benefits. Screening allows detection of BC at an earlier stage as compared to clinical detection through symptoms. Thus, it is reasonable to hypothesize that women with screen-detected cancer experience better quality of life (QoL) than women with clinically detected cancer. This is likely because treatment for women with screen-detected BC is more often less intense, as in this group BC is more often detected in an early stage compared to the group of women with clinically detected BC [4].

Studies on differences in QoL between women with screen-detected and clinically detected BC showed mixed results. For example, a retrospective study from Germany including 735 women with BC revealed that mode of detection was not associated with QoL as measured using the European Organization for Research and Treatment of Cancer Quality of Life Questionnaire (EORTC QLQ) C-30 and BR23 questionnaires [5]. While in Norway, a QoL analysis of 4,487 women using the EuroQol 5-Dimension 5-Level (EQ-5D-5 L) showed that women with screen-detected cancer reported a better QoL than women with symptomatically detected cancer [6]. Furthermore, to the best of our knowledge, there is no study that investigates the QoL of BC patients based on the mode of detection at different time points. Moreover, the magnitude and clinical relevance of QoL differences between women with screen-detected and clinically detected BC have not been adequately researched. Investigating QoL differences between women with screen-detected and clinically detected BC along with its magnitude and clinical relevance may better inform women about the potential impact of screening on QoL.

In the Netherlands, BC screening has been implemented since 1990. Currently, Dutch female residents are offered mammography screening from the age of 50 to 75 every two years. In 2022, the participation rate was 70.7%, which, according to the European guidelines for quality assurance in breast cancer screening and diagnosis, is considered an acceptable level of participation rate [7, 8]. In this study, our objective is to compare QoL between women with screen-detected and clinically detected BC in the target population for screening in the Netherlands. To a large extent, these two groups represent different stages of cancer at diagnosis as a consequence of screening.

## Materials and methods

### Study design and data source

This study is conducted with data from the observational, prospective multicentre ‘Utrecht cohort for Multiple BREast cancer intervention studies and Long-term evaluation’ (UMBRELLA). UMBRELLA includes women with BC or ductal carcinoma in situ prior to surgery or radiotherapy in several hospitals in the Netherlands [9]. Participating hospitals in the UMBRELLA study includes the Utrecht University Medical Centre, Alexander Monro Hospital Bilthoven, St. Antonius Hospital Utrecht, Alrijne Hospital Leiderdorp, and ZGT hospital Twente in The Netherlands. Women who are younger than 18 and/or have limited Dutch language understanding are not eligible to be included in the UMBRELLA cohort [9]. Participants provided consent for the collection and use of clinical data and patient-reported outcomes (PROs) at regular intervals up to 10 years after cohort enrollment [9]. The ethical approval for the UMBRELLA study, registered on clinicaltrials.gov (ID: NCT02839863), was provided by the University Medical Centre Utrecht (NL52651.041.15, Medical Ethics Committee 15/165).

Within UMBRELLA, multiple PROs were measured periodically using validated questionnaires in the Dutch language. In this study, we analysed QoL Questionnaires developed by the EORTC: the EORTC QLQ-C30, supplemented with questionnaire module specifically for BC patients, the EORTC QLQ-BR23 [10, 11]. The EORTC QLQ-C30 is a measure for the assessment of health-related QoL of cancer patients. It consists of multi-item scales and single-item measures related to general health, functional QoL, and symptoms [10]. The EORTC QLQ-BR23 is a validated BC-specific QoL questionnaire module, that has 23 questions covering functional QoL and symptom-related items and scales [11]. For functional and summary scores, a higher score means a better QoL. While for symptomatic scales and items, a higher score indicates greater severity of reported symptoms and should be interpreted as less favourable. The summary score was calculated as the average value of 13 scale and items of the EORTC QLQ-C30 (excluding global QoL and financial difficulties) [12]. This summary score provides a single measure to assess overall QoL [13]. In addition, as depression and anxiety are prevalent among women with BC but not explicitly addressed in the EORTC QLQ-C30 or QLQ-BR23 [14], we used the Hospital Anxiety and Depression (HADS) questionnaire to assess anxiety and depression symptoms [15]. For this study, we analysed all questionnaire results which were completed shortly after diagnosis, mostly prior to treatment (T1), and one-year after treatment (T2).

To evaluate how the questionnaire results might differ between BC patients and the general population, normative scores for the general Dutch female population aged 50 to 75 on EORTC-QLQ-C30, HADS, and sexual-related questions of EORTC-QLQ-BR23 were obtained via the PROFILES (Patient Reported Outcome Following Initial Treatment and Long-term Evaluation of Survivorship) registry (2011 version) [16].

### Participants

In this study, we used data of patients who were recruited within the UMBRELLA cohort between October 2013 and March 2022. The exclusion criteria were: participants with unknown detection mode, unknown clinical information, metastatic stage at diagnosis, men, aged under 50 or above 75, history of tumour in the same breast, and non-participation in the survey during T1 and/or T2.

### Statistical analyses

We stratified eligible BC patients by detection mode, i.e., screen-detected or clinically detected. We calculated the average scores and standard deviations (SD) of all items in the EORTC QLQ-C30, EORTC QLQ-BR23, and HADS separately for T1 and T2. The calculation of each questionnaire scale was performed based on published questionnaire scoring manuals [17, 18]. We also calculated the average score changes between T2 and T1.

The data distribution of each variable was visually examined using Q-Q plots. Despite the existence of variables which were not normally distributed, independent t-tests were used to compare all questionnaire items between women with screen-detected and clinically detected BC. This is because when analysing public health data where the sample size is not small (guidelines suggest minimum of 30 to assume normality according to the central limit theorem), a parametric test, such as independent t-test, is already robust even in non-normally distributed and severely skewed data [19, 20].

For HADS, next to the average score, we also calculated the proportions of women who experienced anxiety symptoms (HADS anxiety scale cut-off > 8), depressive symptoms (HADS depression scale cut off > 8), and who had an indication of a psychiatric disorder (total score cut off > 12). To test the differences of these proportions between women with screen-detected BC and women with clinically detected BC, we performed chi-square tests.

To assess the difference between QoL of women with screen-detected and clinically detected BC, for each item of the questionnaires we calculated the Cohen’s *d*, an index to measure the strength of difference between groups [21]. The difference (irrespective of its positive-negative sign) is classified as trivial (0.0 *≤* *d* < 0.2), small (0.2 *≤* *d < 0.5*), medium (0.5 *≤* *d < 0.8*), or large (*d* *≥* 0.8) [21]. Furthermore, for each QLQ-C30 questionnaire item and scale, we categorized its clinical relevance based on a previously published guideline on interpreting differences of self-reported QoL between different treatment groups [22]. Clinical relevance indicates practical significance or importance of QoL differences within clinical settings. As there are conflicting results from previous studies on the significance of QoL differences among women with BC stratified by detection mode, we expect that there are scales and items which have limited clinical relevance [5, 6]. Thus, the value between trivial and small effect difference based on a previous study was chosen as the clinical relevance cut-off in this analysis [22]. We did not assess clinical relevance of the BR23 and HADS differences because, to our knowledge, there is not yet any published guideline on comparing minimal clinical important difference in outcomes of different treatment groups among cancer patients for these two questionnaires.

## Results

**Fig. 1:**
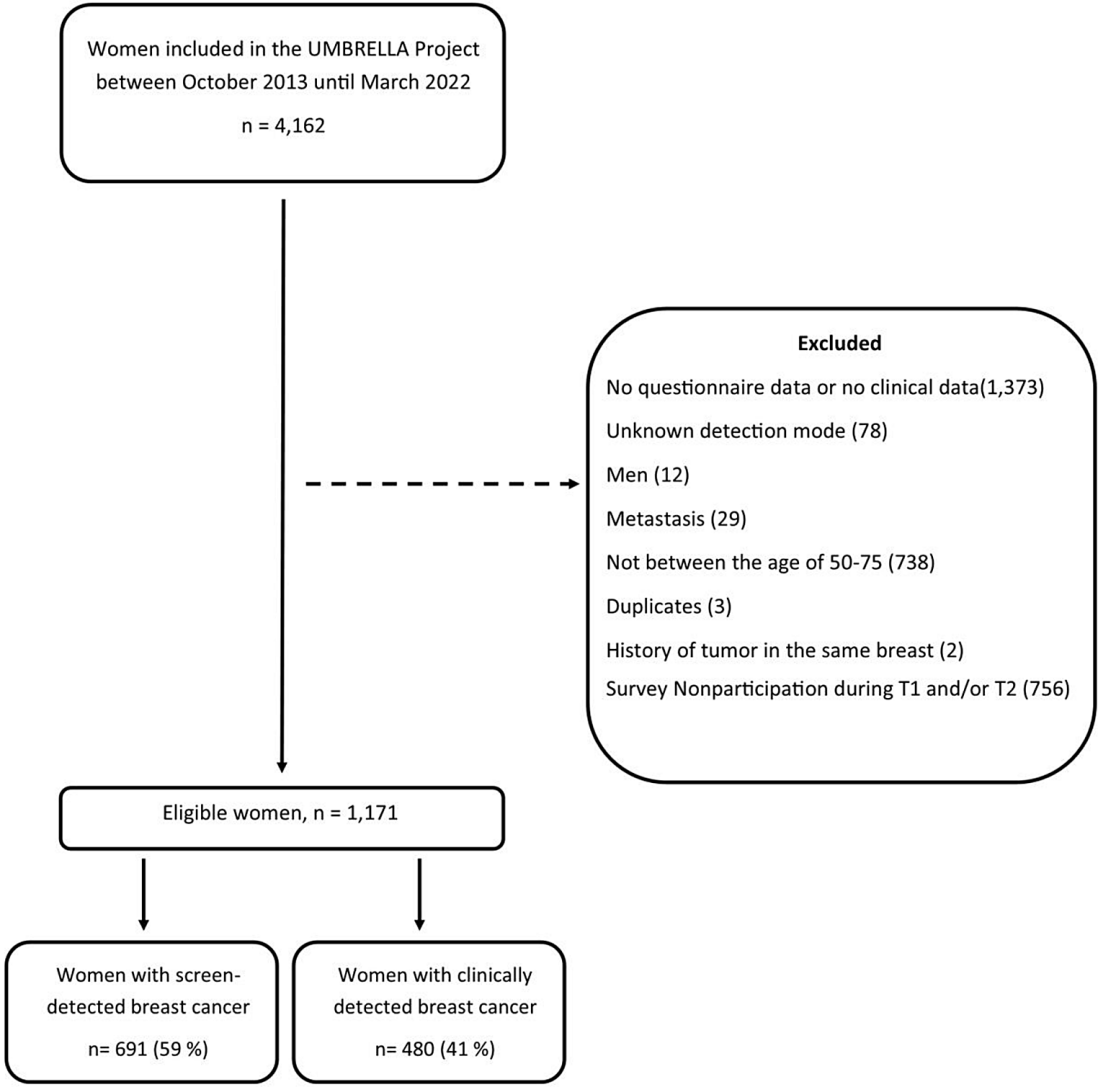
Flowchart illustrating the initial population of women with BC included in the UMBRELLA project, followed by exclusion and classification into screen-detected and clinically detected groups

Between October 2013 and March 2022, there were 4,162 women enrolled in UMBRELLA. Following the exclusion of individuals based on the exclusion criteria, a total of 1,171 women remained, comprising 691 (59%) women with screen-detected BC and 480 (41%) women with clinically detected BC (Fig. 1). Characteristics such as age, stage, surgery, and treatment can be seen in Table 1. Furthermore, we performed 87 independent t-tests and chi-square tests to analyse all questionnaire scales and items. This potentially increases the probability of committing Type 1 errors. Therefore, the *p-*value threshold was adjusted using the Bonferroni correction method, resulting in a *p*-value threshold of 0.00057 (0.05 divided by 87).

### Characteristics of participants

There was a substantial difference (*p* < 0.0001) in stage distribution between women with screen-detected and clinically detected BC, showing that women detected at an earlier stage are more represented in the screen-detected group than in the clinically detected group (Table 1). The combined proportion of Ductal Carcinoma in Situ (DCIS) and Stage 1 cases among women with screen-detected cancer was 75.6%, while it was 45.8% for women with clinically detected cancer.

Furthermore, we found a substantially lower proportion of women who underwent mastectomy among those who were screen-detected (9.3%) as compared to those who were clinically detected (18.8%) (*p* < 0.0001) (Table 1). A smaller proportion of women with screen-detected cancer received neo-adjuvant treatment (7.1%) as compared to women with clinically detected BCs (24.6%) (*p* < 0.0001). Similar results were found for chemotherapy, targeted therapy, and endocrine therapy. The median time interval between study inclusion and the baseline survey was 13 days for women with screen-detected cancers and 14 days for women with clinically detected cancers.

**Table 1:** Characteristics of women with BC (*n* = 1,171) stratified by mode of detection

	Screen-detected		Clinically detected		*p*-value
	*n* = 691	59.0%	*n* = 480	41.0%	
**Age**					
50–54	164	23.7%	127	26.5%	0.0057
55–59	124	17.9%	120	25.0%	
60–64	151	21.9%	88	18.3%	
65–69	159	23.0%	81	16.9%	
70–75	93	13.5%	64	13.3%	
**Tumour stage**					
DCIS	144	20.8%	28	5.8%	< 0.0001^**^
Stage I	379	54.8%	192	40.0%	
Stage II	151	21.9%	200	41.7%	
Stage III	17	2.5%	60	12.5%	
**Most-invasive surgery**					
Unknown	0	0.0%	1	0.2%	< 0.0001^**^
Lumpectomy	627	90.7%	389	81.0%	
Mastectomy	64	9.3%	90	18.8%	
**Neo-adjuvant treatment**	49	7.1%	118	24.6%	< 0.0001^**^
Adjuvant Therapy*					
Chemotherapy	116	16.8%	138	28.8%	< 0.0001^**^
Targeted therapy	40	5.8%	60	12.5%	< 0.0001^**^
Endocrine therapy	262	37.9%	261	54.4%	< 0.0001^**^

^*^A patient can receive more than one type of adjuvant therapy

^**^Statistically significant based on Bonferroni corrected *p*-value threshold of 0.00057

### Quality of life differences between mode of detection

In general, women with screen-detected BC had more favourable scores on questionnaires measuring QoL than women with clinically detected cancers (Tables 2 and 3). Score differences in QoL between women with screen-detected and women with clinically detected BC were found in both the initial survey (T1) and the follow-up survey (T2).

At T1, women with screen-detected BC showed statistically significantly better scores on three scales and items of the QLQ-C30 questionnaire, namely fatigue, appetite loss, and the summary score, compared to women with clinically detected cancer (Table 2). Additionally, the score differences of four scales and items in the QLQ-BR23 (body image, future perspective, side effects of systemic therapy, and arm symptoms) between women with screen-detected and clinically detected BC were also statistically significant, favouring women with screen-detected BC (Table 3).

At T2, women with screen-detected BC showed statistically significantly better scores across seven scales and items of the QLQ-C30 questionnaire, namely general health, physical functioning, cognitive functioning, social functioning, fatigue, constipation, and the summary score, compared to women with clinically detected BC (Table 2). In the QLQ-BR23 questionnaire at T2, the score differences of body image, side effects of systemic therapy, breast symptoms, and arm symptoms, also showed statistical significance in favour of women with screen-detected BC (Table 3). There is no scale of the HADS questionnaire where the difference in scores is statistically significant at T1 and/or T2. Thus, at T2, statistically significant score differences between women with screen-detected and clinically detected BC were found on more questionnaire scales and items than at T1.

The difference (Cohen’s *d*) of all questionnaire scales and items were in the range of 0.00 to 0.39, irrespective of the positive or negative sign. For example, Cohen’s *d* value for sexual functioning at T1 was 0.04 in favour of women with screen-detected BC suggesting a trivial difference (Table 3). While also at T1, the Cohen’s *d* value for systemic therapy was 0.39 in favour of women with screen-detected BC, indicating a small difference. These findings indicate that in general, the QoL differences between women with screen-detected and clinically detected BC can be considered either trivial or small.

Regarding the clinical relevance of score differences in EORTC QLQ-C30 scales and items, there were almost no clinically relevant score differences between detection modes at T1 (Table 2). The only exception was the score difference in emotional functioning, which was considered to have limited clinical relevance in favour of women with screen-detected BC. At T2, average score differences for general health, emotional functioning, fatigue, and constipation between screen-detected and clinically detected group were considered to have small clinical relevance in favour of women with screen-detected BC [22].

**Table 2:** EORTC QLQ-C30 questionnaire scales and items compared between women with screen-detected and clinically detected BC measured in the initial survey (T1) and 12 months post-treatment follow-up survey (T2). Population normative values were obtained from the PROFILES study [15]

	Initial Survey (T1)				Follow-up survey (T2)				ΔT1^†^	ΔT2^†^	CR^††^	Normative values^§^
	Screen-detected	Clinically detected	*p*-value	Cohen’s *d*	Screen-detected	Clinically detected	*p*-value	Cohen’s *d*				Mean (sd)
	Mean (sd)				Mean (sd)							
**QLQ-C30**												
Summary Score	86.1 (11.0)	83.0 (12.3)	< 0.0001^*^	0.14	88.3 (11.3)	84.6 (13.4)	< 0.00001^*^	0.30	3.1	3.7	n/a	88.0 (12.1)
General health	76.7 (17.0)	73.7(17.7)	0.00399	0.17	80.4 (16.4)	76.2 (18.4)	0.00006^*^	0.25	3.0	4.2 ^α^	4	75.2 (17.8)
Functional QoL												
*Physical functioning*	87.9 (14.4)	85.5 (15.7)	0.00725	0.16	88.8 (14.2)	85.6 (15.9)	0.00046^*^	0.21	2.4	3.2	5	85.4 (16.8)
*Role functioning*	75.3 (26.2)	70.7 (26.7)	0.00321	0.18	84.8 (22.7)	80.4 (24.9)	0.00252	0.18	4.6	4.4	6	83.2 (23.9)
*Cognitive functioning*	86.5 (18.1)	83.3 (20.4)	0.00602	0.16	86.0 (18.9)	80.2 (22.0)	< 0.00001^*^	0.29	3.2	5.8	n/a	90.7 (16.7)
*Emotional functioning*	80.1 (17.8)	77.0 (20.4)	0.00759	0.16	85.7 (17.7)	82.1 (20.1)	0.00149	0.20	3.1^α^	3.6^α^	3	85.1 (18.1)
*Social functioning*	85.5 (20.0)	83.2 (20.6)	0.05221	0.12	91.0 (17.2)	86.1 (20.6)	0.00002^*^	0.26	2.3	4.9	5	91.7 (17.5)
Symptoms												
*Fatigue*	24.7 (21.4)	29.2 (22.4)	0.00054^*^	-0.21	20.8 (20.4)	27.5 (23.6)	< 0.00001^*^	-0.31	-4.5	-6.7 ^α^	5	20.7 (21.3)
*Nausea, vomiting*	2.9 (8.4)	5.0 (11.6)	0.00099	-0.21	3.0 (9.8)	4.6 (11.1)	0.00918	-0.16	-2.1	-1.6	3	3.9 (11.3)
*Pain*	18.9 (21.2)	21.4 (22.9)	0.05639	-0.11	13.5 (20.3)	17.7 (22.4)	0.00115	-0.20	-2.5	-4.2	6	20.7 (23.4)
*Dyspnoea*	8.0 (18.0)	10.9 (20.1)	0.01128	-0.15	12.0 (21.2)	13.8 (22.7)	0.17081	-0.08	-2.9	-1.8	4	8.7 (18.6)
*Insomnia*	25.6 (27.1)	29.3 (29.5)	0.02474	-0.13	24.3 (27.5)	26.6 (28.3)	0.15656	-0.08	-3.7	-2.3	4	22.3 (28.1)
*Appetite loss*	5.8 (16.5)	9.9 (20.4)	0.00023^*^	-0.23	4.7 (15.9)	6.0 (15.9)	0.20506	-0.07	-4.1	-1.3	5	3.7 (12.3)
*Constipation*	6.9 (17.7)	9.7 (18.7)	0.01183	-0.15	6.5 (16.1)	11.5 (21.8)	0.00002^*^	-0.27	-2.8	-5.0 ^α^	5	7.4 (17.9)
*Diarrhoea*	4.4 (12.9)	6.5 (15.8)	0.01651	-0.15	4.3 (13.0)	5.4 (13.9)	0.18580	-0.08	-2.1	-1.1	3	4.8 (14.8)
Financial difficulties	2.8 (11.5)	5.4 (15.5)	0.00214	-0.19	4.9 (13.9)	7.2 (18.1)	0.02151	-0.15	-2.6	-2.3	3	4.6 (15.4)

^†^Score difference between screen-detected and clinically detected.

^††^CR= Clinical relevance. This column displays chosen cut-off values taken from a previous study to determine minimum score difference for each scale and item to be considered clinically relevant. The value between trivial and small effect difference was chosen as the cut-off point (summary score and cognitive functioning do not have cut-off value based on the previous study) [22]

^§^Population norm QoL values were obtained from the PROFILES study [16]

^*^Statistically significant based on Bonferroni corrected *p*-value threshold of 0.00057

^α^Clinically relevant

**Table 3:** EORTC QLQ-BR23 and HADS questionnaire scales and items compared between women with screen-detected and clinically detected BC measured in the initial survey (T1) and 12 months post-treatment follow-up survey (T2). Population normative values were obtained from the PROFILES study

	Initial Survey (T1)				Follow-up survey (T2)				Normative valuesdifference betwe	
	Screen-detected	Clinically detected	*p*-value	Cohen’s *d*	Screen-detected	Clinically detected	*p*-value	Cohen’s *d*		
	Mean (sd)				Mean (sd)				Mean (sd)	
**QLQ-BR23**										
Functional QoL										
*Body image*	90.8 (15.3)	86.4 (18.2)	< 0.0001^*^	0.27	92.1 (14.8)	88.0 (18.7)	0.00009^*^	0.25	n/a	
*Sexual functioning*	18.7 (19.2)	18.0 (18.6)	0.54175	0.04	23.6 (19.8)	22.4 (19.4)	0.35755	0.06	25.3	(23.8)
*Sexual enjoyment*	56.5 (24.3)	56.5 (25.5)	0.98235	0.00	60.3 (27.4)	56.3 (29.5)	0.11337	0.14	63.7	(27.9)
*Future perspective*	67.6 (23.1)	62.0 (26.6)	0.00017^*^	0.23	74.8 (22.3)	70.1 (23.7)	0.00063	0.21	n/a	
Symptoms										
*Systemic therapy side effect*	9.2 (10.3)	13.7 (13.8)	< 0.0001^*^	-0.39	12.5 (12.2)	15.9 (12.7)	0.00001^*^	-0.27	n/a	
*Upset due to hair loss*	17.2 (23.9)	23.6 (28.3)	0.15514	-0.24	19.6 (26.7)	19.4 (23.6)	0.95846	0.01	n/a	
*Breast symptom(s)*	22.3 (17.8)	22.8 (17.5)	0.63709	-0.03	14.8 (15.5)	18.9 (17.8)	0.00005^*^	-0.25	n/a	
*Arm symptom(s)*	12.2 (14.9)	16.0 (17.7)	0.00014^*^	-0.24	10.1 (14.6)	16.3 (19.1)	< 0.00001^*^	-0.37	n/a	
**HADS**										
Total score	8.2 (5.8)	8.8 (6.5)	0.11131	-0.21	7.2 (5.5)	8.2 (6.5)	0.00197	-0.19	8.2	(6.2)
Total score (cut-off > 12)	20.4%	20.9%	0.82906		15.1%	21.0%	0.00931		20.5%	
*Anxiety symptoms*	5.2 (3.3)	5.4 (3.6)	0.18605	-0.08	4.3 (3.0)	4.8 (3.4)	0.00635	-0.16	4.3	(3.5)
*Anxiety symptoms* (cut-off > 8)	14.1%	16.7%	0.23230		9.23%	14.1%	0.01029		13.3%	
*Depression* symptoms	3.0 (3.0)	3.3 (3.4)	0.11221	-0.10	2.8 (3.0)	3.4 (3.5)	0.00217	-0.19	3.9	(3.4)
*Depression* symptoms (cut-off > 8)	6.7%	8.9%	0.16968		6.6%	10.1%	0.03175		12.9%	

^*^Statistically significant based on Bonferroni corrected *p*-value threshold of 0.00057

^§^Population norm QoL values were obtained from the PROFILES study (For BR23 questionnaire, only sexual-related functional scales are available) [16]

Using the *p-*value threshold of 0.00057, we found no statistically significant difference in questionnaire score change between initial (T1) and follow-up (T2) survey among women with screen-detected and clinically detected BC. (Table 4).

**Table 4:** Questionnaire score changes between initial and follow-up survey compared between women with screen-detected and clinically detected BC

	Changes between initial (T1) and follow-up survey (T2)			
	Screen-detected	Clinically detected	*p*-value	Cohen’s *d*
	Mean (sd)			
**QLQ-C30**				
Summary Score	2.2 (11.0)	1.8 (17.4)	0.503	0.04
Global health	3.9 (17.8)	2.5 (18.4)	0.113	0.07
Functional QoL				
*Physical functioning*	0.9 (13.6)	0.2 (14.3)	0.407	0.05
*Role functioning*	9.5 (27.5)	10.0 (29.5)	0.771	-0.02
*Cognitive functioning*	-0.4 (18.8)	-2.9 (20.3)	0.034	0.01
*Emotional functioning*	5.6 (18.9)	5.1 (19.5)	0.653	0.03
*Social functioning*	5.3 (21.6)	3.1 (22.7)	0.094	0.10
Symptoms				
*Fatigue*	-4.0 (22.0)	-1.9 (22.1)	0.125	-0.09
*Nausea, vomiting*	-0.01 (11.9)	-0.4 (14.5)	0.602	0.03
*Pain*	-5.4 (23.9)	-3.8 (24.5)	0.280	-0.07
*Dyspnoea*	4.0 (20.2)	2.6 (22.8)	0.291	0.07
*Insomnia*	-1.5 (28.1)	-2.6 (22.8)	0.534	0.04
*Appetite loss*	-1.1 (18.6)	-4.0 (21.8)	0.017	0.15
*Constipation*	-0.3 (21.0)	1.7 (22.3)	0.125	-0.09
*Diarrhoea*	-0.05 (16.4)	-1.2 (18.7)	0.307	0.06
Financial difficulties	2.1 (14.3)	1.8 (17.4)	0.731	0.02
**QLQ-BR23**				
Functional QoL				
*Body image*	1.3 (15.7)	1.9 (16.9)	0.545	-0.04
*Sexual functioning*	5.0 (18.2)	4.2 (17.9)	0.478	0.05
*Sexual enjoyment*	5.0 (25.1)	0.5 (27.1)	0.130	0.17
*Future perspective*	7.3 (24.7)	8.1 (22.8)	0.581	-0.03
Symptoms				
*Side effects of systemic therapy*	3.3 (12.7)	2.0 (13.8)	0.145	0.09
*Sad due to hair loss*	2.7 (27.1)	0.0 (30.2)	0.763	0.09
*Breast symptom(s)*	-7.7 (20.2)	-4.1 (21.1)	0.004	-0.18
*Arm symptom(s)*	-2.3 (17.1)	0.01 (20.0)	0.046	-0.12
**HADS**				
Total score	-1.1 (5.3)	-0.6 (5.3)	0.131	-0.09
*Anxiety symptoms*	-0.8 (3.0)	0.6 (2.9)	0.270	-0.07
*Depression symptoms*	-0.3 (3.3)	0.0 (3.1)	0.118	-0.09

^*^Statistically significant based on Bonferroni corrected *p*-value threshold of 0.00057

## Discussion

### Quality of life differences between mode of detection

The results of this study showed that women with screen-detected BC reported a better and statistically significant QoL compared to women with clinically detected BC up to one year post treatment. However, the difference is minimal (Cohen’s *d* between 0.00 and 0.39) and the clinical relevance of the difference is limited. We also found that self-reported QoL differences according to detection mode are more prominent one year after treatment than shortly after diagnosis.

The better QoL reported by women with screen-detected cancer may be explained by a higher proportion of screen-detected cancers detected at earlier stages. The difference in stage at detection affects treatment choices [23, 24], and less invasive treatment may result in a better QoL. Based on a previous study, women with less invasive breast-conserving therapy reported better QoL than women who underwent mastectomy [25]. Another study that observed women with BC reported that chemotherapy and endocrine therapy have a detrimental impact on QoL, with the impact of endocrine therapy that persisted for at least 2 years after diagnosis [26]. Post-menopausal patients who received endocrine therapy also reported lower QoL [27]. Another important consideration when evaluating QoL of women with BC according to mode of detection is that screening detects BCs at an earlier stage and age. Without screening, women diagnosed with BC through screening in this study might have been diagnosed clinically at a later time, very likely at an older age. Therefore, we did not adjust for age and stage in this study as they are intermediary variables rather than confounders.

There were more differences and more statistically significant differences between the two modes of detection at T2 than at T1. The difference in the summary score of QLQ-C30 between detection modes was also larger at T2 as compared to T1 (Table 2). Looking into the scores of scales and items which differed and were statistically significant, it appears that at T2, there was an improvement of functional QoL and reduced symptoms experienced by BC patients in both groups. These findings implied that the impact of detecting BC earlier on QoL is larger at one year after treatment than shortly after diagnosis. While previous studies have shown QoL differences between mode of detection at a single time point of measurement [5, 6], our study is the first to show QoL differences between women with screen-detected and clinically detected BC at two different time points, shortly after diagnosis and one year after treatment.

At T1, there was no statistically significant difference in QLQ-C30 functional QoL scale or item between the two groups (Table 2). However, the score difference between mode of detection for one functional item, namely body image, and one functional scale, future perspective, of QLQ-BR23 were statistically significant at T1 (Table 3). This shows that the QLQ-BR23 may be more sensitive at detecting differences in functional QoL between screen-detected and clinically detected BC patients as compared to the QLQ-C30. This can be expected as the QLQ-BR23 questionnaire is tailored specifically to BC patients, whereas the QLQ-C30 which aims to assess QoL of cancer patients in general [10, 11]. Therefore, the way questions are formulated in the QLQ-BR-23 might better capture the BC-specific circumstances experienced by the patients.

Although statistical differences in QoL between modes of detection were found using the QLQ-C30 as early as shortly after diagnosis, the clinical relevance of these QoL differences is limited. Score differences on the emotional functioning scale between the screen-detected and clinically detected groups were found to be clinically relevant at both time points, shortly after diagnosis and one-year post-treatment. In contrast, score differences on other clinically relevant scales and items, such as general health, constipation, and fatigue, were only observed one-year post-treatment. For clinicians, these findings may contextualize statistical results and help identify the specific aspects of QoL that differ between screen-detected and clinically detected BC patients in clinical settings.

The inclusion of the HADS questionnaire in this study provided valuable insights into the psychological well-being of the participants. While there was no statistically significant difference in HADS scores between the two groups, it is noteworthy that women with screen-detected cancer consistently reported fewer symptoms of anxiety and depression (Table 3). This finding is consistent with a previous study from Ireland [28]. Another noteworthy finding is that at T2, the proportion of women experiencing anxiety and depressive symptoms seemed to be generally lower than at T1.

We did not perform statistical tests comparing the scores of women with BC detected by both detection modes and the scores of the general populations as it is not the aim of this study. Thus, whether the scores are different and statistically significant remains unknown. However, looking at the average of scores alone, we found no specific pattern in how the self-reported QoL among BC patients (regardless of its mode of detection) compared to the general population. For example, women with screen-detected and clinically detected BC appeared to have lower EORTC-QLQ-C30 summary score than the general population at T1 (86.1, 83.0 and 88.0 respectively) (Table 2). While at T2, the average summary score for women with screen-detected BC seemed to be improved (88.3), making it appear to be slightly higher than the general population (88.0). Another example is the score for physical functioning (Table 2). At T1, both groups—women with screen-detected and clinically detected cancer—appeared to have higher scores than the general population (85.4), with the scores of 87.9 and 85.5 respectively. Similarly, at T2, the scores appeared to remain higher at 88.8 and 85.6 respectively.

In some questionnaire items and scales, women with BC even showed more favourable scores as compared to its corresponding normative values. This might be caused by positive feelings experienced by women with BC since they managed to survive so far. Women may also value their QoL differently after being diagnosed with and surviving cancer. Another possible cause is that BC is more prevalent in women with higher socioeconomic status and women with higher socioeconomic status tend to report better QoL [29, 30]. Additionally, it may also be caused because healthier patients or patients who had fewer symptoms were more likely to participate in this survey. Although this kind of selection might occur in both groups—women with screen-detected and clinically detected BC.

### Strengths and limitations

The strength of this study is that the QoL was measured at two different time points. This makes it possible to capture the QoL dynamics experienced by women with screen-detected and clinically detected BC up to one year after treatment. Secondly, we complemented the EORTC QLQ-C30 with a questionnaire module specifically for BC patients, the QLQ-BR23. In addition, we also used of HADS questionnaire to assess depression and anxiety symptoms among BC patients. This approach allows a comprehensive assessment of QoL among women with BC considering both physical and psychological aspects.

Our inclusion period was between October 2013 and March 2022, which means it included the period of the COVID-19 pandemic. This might raise concern on how the pandemic affected the results of this study. In the Netherlands, the national BC screening programme was stopped between March 2020 until June 2020 due to COVID-19 pandemic. A relevant study examining the pandemic’s impact on BC diagnosis and treatment in the Netherlands found that while there was a reduction in the incidence of lower-stage diseases, treatment delays were limited to the first eight weeks of the pandemic. Patients diagnosed thereafter experienced no significant delays in their initial treatments, although there was a notable shift in treatment strategies, with an increased use of primary hormonal treatments instead of surgical options [31]. Moreover, any delay and change on diagnostics and treatment might affect all women recruited during the pandemic, regardless of screen-detected or clinically detected. Furthermore, the majority of women included in this study were recruited before the pandemic. Therefore, we assume that the impact of the pandemic on the results of this study is minimal.

Certain biological aspects may not be fully captured by this study. Evidence suggests that women with Human Epidermal Growth Factor Receptor 2 (HER2) and Triple Negative BC (TNBC) are less frequently screen detected. TNBC is challenging to detect on mammography as it often lacks detectable features [32]. Both types are aggressive and may require intensive treatment, potentially impacting QoL [33, 34]. Therefore, having screen-detected does not always correlate with better QoL, as BC subtypes like TNBC and HER2-positive are less likely to be found through screening. Additionally, The Bonferroni correction method in this study resulted in a more conservative *p*-value threshold (0.00057) than the traditionally chosen *p*-value threshold (0.05). While this approach has minimized the probability of committing a Type I error, the probability of having a Type II error is increased [35]. Thus, it is possible that there are actually more questionnaire items which are statistically significantly different between women with screen-detected and clinically-detected BC. The result of QLQ-C30 emotional functioning item at T2 (Table 2) which showed clinical relevance, but no statistical significance may indicate a Type II error. Nevertheless, the main result of this research will largely remain unaffected, indicating that the magnitude and clinical relevance of the QoL difference between the two detection modes are marginal, regardless of the number of scales or items displaying statistically significant score differences.

## Conclusions

In the target population for screening in The Netherlands, we found that women with screen-detected BC reported a better and statistically significant QoL than women with clinically detected BC. The QoL differences were larger at one-year after treatment rather than shortly after diagnosis. However, the magnitude of the differences and clinical relevance of these QoL differences are limited. Our findings add to the current knowledge on the impact of the mode of detection on QoL after BC treatment. This may allow women to make better-informed decisions regarding BC screening participation.

## Data Availability

The data that support the findings of this study are available upon reasonable request and such request should be addressed to Prof Dr HM Verkooijen.

## Declarations

### Originality of the work

This manuscript is not under consideration for publication elsewhere.
